# On the crashworthiness analysis of bio-inspired DNA tubes

**DOI:** 10.1038/s41598-024-59258-2

**Published:** 2024-04-12

**Authors:** Amir Najibi, Liwen Zhang, Dongli Zheng

**Affiliations:** 1 School of Automotive Engineering, Hubei University of Automotive Technology, No. 167, Checheng West Road, Shiyan, 442002 Hubei China; 2 Hubei Key Laboratory of Automotive Power Train and Electronic Control, Shiyan, 442002 China; 3 Faculty of Mechanical Engineering, Semnan University, Semnan, Iran

**Keywords:** Bio-inspired DNA tube, SEA, IPF, Quasi-static, Numerical simulation, Aerospace engineering, Civil engineering, Mechanical engineering

## Abstract

This study presents a thorough numerical evaluation of the crashworthiness properties of a new bio-inspired DNA tubes (BIDNATs) with circular, elliptical, and rectangular cross-sections. Deformation and crashworthiness behaviors are evaluated using axial quasi-static crushing simulations by ABAQUS/Explicit (Abaqus 6.14, https://www.3ds.com/products-services/simulia/products/abaqus/). The study compares the performance of conventional tubes with rectangular and elliptical cross-sections to DNA-inspired tubes. Increasing the rotation angle leads to more helices and a pronounced helix angle, resulting in lower initial peak force (IPF). However, lower cross-section aspect ratios generally have higher IPF and specific energy absorption (SEA) values. BIDNATs with rectangular cross-sections and a 540° rotation angle have the lowest SEA and IPF values across all aspect ratios. Notably, for the 110/100 aspect ratio, the SEA of E110/100 is 71% higher than the conventional tube. Overall, BIDNATs with elliptical cross-sections and a 360° rotation angle exhibit higher SEA values and lower IPF values, particularly for a width (W) of 100 mm. Conventional circular and elliptical tubes generally have SEA values exceeding 6 J/g, with only E110/100 surpassing this among DNA-inspired tubes. The NE110/100 tube has the highest SEA, surpassing E110/100 by 54%, while its IPF is 10% greater than DNA-inspired E110/100. It's worth noting that conventional circular and elliptical tubes have higher IPF values compared to their DNA-inspired counterparts. These findings offer valuable insights for engineers and researchers in the design of crash tubes to improve overall vehicle safety for both occupants and pedestrians.

## Introduction

Thin-walled metallic constructions have been extensively employed in the automotive, railroad, aircraft, and shipbuilding sectors due to their high energy absorption and remarkable lightweight qualities^1,2^. High kinetic energy from an impact is anticipated to be completely absorbed by energy-absorbing components through significant plastic deformation. This has led to the development of a wide variety of energy absorbers with a variety of various structural shapes, including simple and cone tubes^1,3,4^, grooved tubes^5–7^, combined tubes^8–10^, the origami tubes^11,12^, and Auxetic materials/structures^13–15^. The two simplest and most popular tubular shapes for energy absorption are regarded as circular and square cross sectional tubes. Two popular theoretical models for a circular tube were first suggested by Alexander^16^ and Pugsley and Macaulay^17^ in 1960. These theories have been improved by Grzebieta^18^, Wierzbicki et al.^19^, and Singace et al.^20^. Consequently, Abramowicz and Jones created the traditional theoretical models for the modes of circular and square tubes^21^. Square tubes have drawn a lot of interest as an energy absorber because, in comparison to the circular tubes, they are significantly simpler to attach to other structural parts and have lower initial peak forces^2^.

Over the years, thin-walled tubes have evolved from hollow, smooth walls to grooved and paper-folded forms, which have greatly enhanced their impact resistance. Given that the impact resistance of a metal thin-walled tube depends primarily on the dissipation of plastic energy and the initial peak force, having grooves and pre-folded paper is advantageous for improving impact resistance.

Abramowicz and Wierzbicki conducted experimental and theoretical research on multi-cornered mild steel tubes^22^, which showed that the mean crushing force of hexagonal tubes was greater than that of square tubes with the same mass. Later, other concave-section tubes were also suggested, including star and criss-cross tubes, which were demonstrated to deform more steadily and absorb more energy^23,24^. Li et al. conducted a study on the multi-objective optimization of sinusoidal cross-sections to improve the axial crashworthiness of double-hat tubes^25^. Another method for increasing the number of corners is the multi-cell arrangement, which outperforms conventional tubular absorbers in terms of energy absorption characteristics^26–29^. Chen and Wierzbicki's simplified super folding element (SSFE) approach was used to develop a traditional analytical solution for the mean crushing force of multi-cell tubes^29^, which Zhang and the team further updated the approach^30,31^.

Sun et al.^32^ were the first to do the crashing analysis and multi-objective optimization for thin-walled structures with axially graded thickness. Later, Zhang et al.^33^ conducted an experimental and numerical investigation on a tube with lateral graded thickness. A comprehensive numerical investigation on the axial crushing of multi-cell tubes with graded wall thickness was carried out by Fang et al.^34^, and the results showed that the thickness gradient in various places may significantly impact the crashing behavior of the graded multi-cell tubes. These studies showed that the axial gradient could significantly lower the initial peak crushing force while the lateral gradient could significantly increase the SEA of multi-cell tubes^35,36^. In addition, functionally graded geometry multi-cell tubes in which the size of the cells has changed gradually along with the lateral directions under axial crushing has been investigated by^37^. The improvement of the SEA due to the local buckling effect of the lateral cell geometry gradations is one of the advantageous of the proposed structure.

Moving from a hollow to a foam-filled design is another development tendency in thin-walled tubular structures^38^. Different types of the lightweight porous material as foams can be used to enhance the crashworthiness of the tubular structures during uni-axial and multi-axial crushing^39^. Yu et al. recently performed an experimental investigation of the static and dynamic axial crushing characteristics of an aluminum density-graded foam-filled tube^40^. In the most of the studies the interactions of foam and tube walls have been considered ideal and adding foam to the thin-walled tube increases the initial peak force that is undesirable for passenger cars; however, it increases the SEA^38^. Furthermore, the plate-lattice structural material-filled multilayer square tube exhibited significantly higher strength and specific energy absorption compared to the foam-filled tube^41^.

It is well known that structures with excellent energy absorption capabilities play a crucial role in impacts, and in engineering applications. Biomimetic structures, inspired by many biological structures in nature, have been shown to provide significant improvements in energy absorption capacity over conventional structures^25,42^. This has led people to develop a number of bio-inspired energy absorbers over time^43,44^. In nature, plant stems have unique morphologies that enable them to support weight, withstand wind loads, and effectively adapt to their surroundings. For instance, several creative bio-inspired multi-cell constructions were presented by Liu et al.^45^, Huang et al.^46^, Hu et al.^47^ and Deng et al.^48^ by imitating the traits of the stems of palm and bamboo trees. Gong et al.^49^ proposed a different multi-cell tube that resembles plant stems and showed improved crashworthiness performance over conventional bi-tubular tubes. The improvement and reduction of the SEA and IPF were achieved through the introduction of a novel hierarchical gradient structure called the hexagon hierarchical gradient structure (HHGS), proposed by Chen et al.^50^, when subjected to oblique loads. Due to its low weight and tremendous strength, the forewing of a beetle is another remarkable bio-structure^42,51^. Because of this, several complex multi-cell energy absorbers have been developed^46,52^ that imitate certain essential structural elements of beetle forewings.

Animal bones have been a significant source of bio-inspiration due to their unique characteristics for heavy load bearing. Therefore, certain cutting-edge multi-cell tubular components that demonstrate obvious advantages over the traditional thin-walled tubes have been proposed by imitating the graded architectures of animal bones^37,53,54^. Recently, an intriguing crash box that resembles the human femur, consists of an inner core packed with materials having a negative Poisson's ratio and an exterior shell that is concave, was created^55^. Furthermore, energy absorption characteristic of the multi-cell functionally graded aluminum foam-filled with graded thickness inspired from the human femur has been investigated by^56^.

Even though the preceding research showed several intriguing tubular energy absorbers, these structures have yet to be adequately investigated, hinting that their potential and capacity might be further explored. Through adaption to varied severe situations, biological systems such as plants and animals have created several unique bio-structures with high energy absorption and lightweight qualities. Over time, this has led humans to develop a variety of bio-inspired energy absorbers. Nature presents numerous inspiring examples of tubular structures with low density, great strength, and high energy absorption capacities, which inspire the construction of unique tubular structures with exceptional energy absorption capabilities^57^. Therefore, this work suggests a new tubular energy absorber called the bio-inspired DNA tube (BIDNAT), which is made up of the revolution of the inspired DNA helices. Quasi-static axial crushing and systematic numerical simulations are used to evaluate the deformation behavior and crashworthiness characteristics. The research is expected to broaden the bio-inspiration approach for creating some innovative energy absorption structures.

## Structural design and analysis

**Figure 1:**
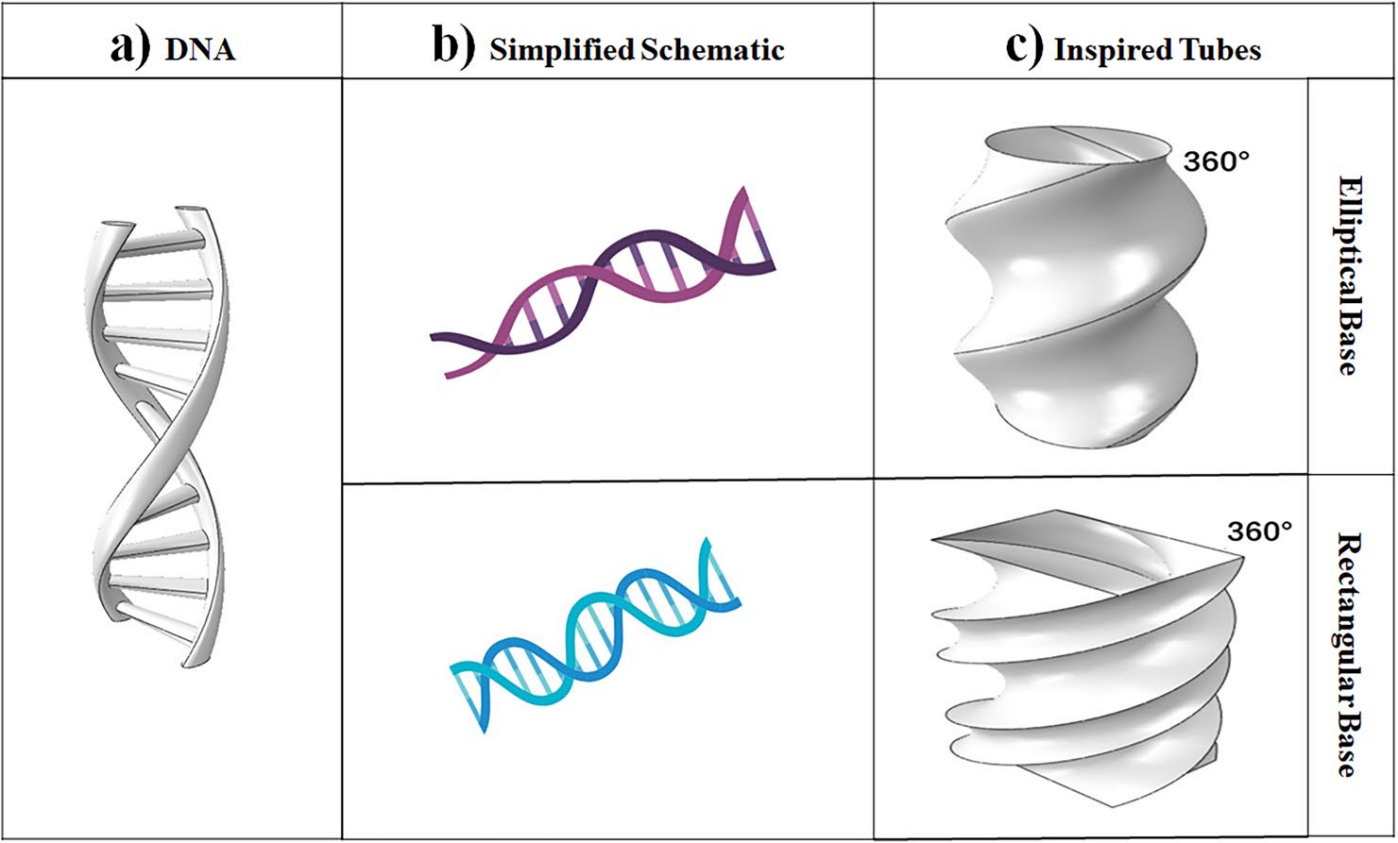
Bio-inspired DNA tubes (BIDNATs). (**a**) Real DNA [created by SOLIDWORKS 2016 (www.solidworks.com)], (**b**) the simplified scheme [created by Biorender (https://biorender.com/)] and (**c**) inspired tubes with rotation angle of 360° [created by SOLIDWORKS 2016 (www.solidworks.com)].

This study aims to examine the energy absorption properties of the bionic DNA thin-walled tube. To achieve this, bionic DNA, which possesses a unique microstructure found in nature^58^, is utilized. Figure 1 shows the bionic shape of DNA, which serves as a model for this investigation. The study focuses on analyzing three different cross-sections: circular, elliptical, and rectangular. The width of the cross-section is varied while keeping the length constant, and vice versa. Furthermore, the bionic structure is employed to create the structural design of the thin-walled tube under different conditions, including two rotational angles (360° and 540°).

The tube models were precisely constructed using SolidWorks (SOLIDWORKS 2016, www.solidworks.com) software, incorporating three distinct cross-sections, varying dimensions within each cross-section, and different rotation angles. To simulate the crashworthiness behaviors, the ABAQUS/Explicit (Abaqus 6.14, https://www.3ds.com/products-services/simulia/products/abaqus/) finite element software was employed. Energy-absorbing tubes were designed for rectangular and elliptical cross-sections by manipulating the width while keeping the length constant, as well as by altering the length while maintaining the width. These crafted designs pave the way for further scientific exploration and analysis in the realm of energy absorption properties.

### Tubes with circular cross-sections

**Figure 2:**
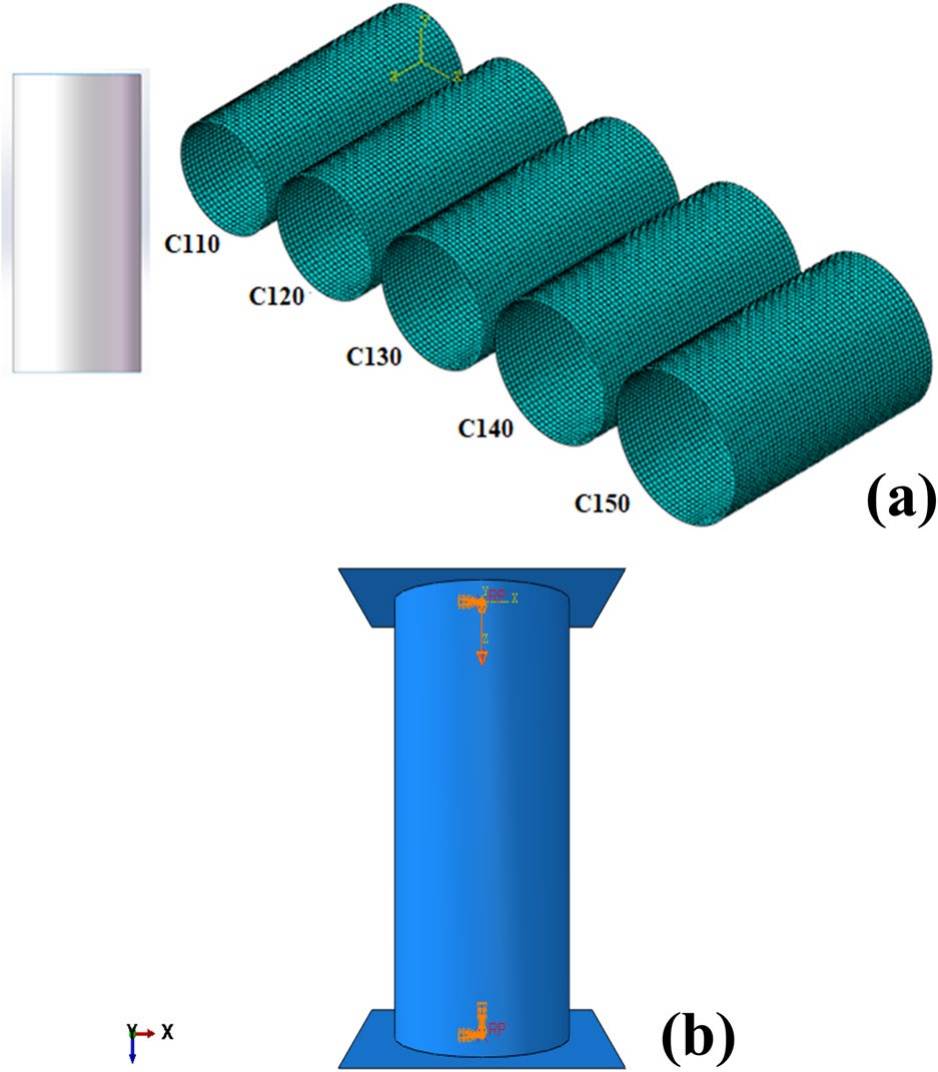
Finite element models. (**a**) Five circular section tubes and [created by SOLIDWORKS 2016 (www.solidworks.com)] and (**b**) the simulation model of a circular cross-section tube with boundary conditions [created by Abaqus 6.14, (https://www.3ds.com/products-services/simulia/products/abaqus/)].

For the sake of comparison the circular cross-section of the tube has been chosen (Fig. 2a). Circular tubes have been found to have good SEA, thanks to their relatively uniform stress distribution and resistance to bending and buckling. In addition, the circular shape also allows for efficient space utilization and easier manufacturing processes, making them a popular choice in many industries^59^. However, the crashworthiness of circular tubes can still be further improved by optimizing their configuration. Consequently, the model shown in Fig. 2a was built from five different sets of arbitrary radius dimensions.

In the nomenclature of circular cross-section tubes, such as C110, the prefix "C" signifies the specific shape of a circular cross-section, while the numerical value "110" denotes the diameter of the circle. This naming convention can be universally applied to designate circular tubes with different diameters. It is important to highlight that, in order to maintain consistency and facilitate practical application, the height of all the tubes has been standardized at 250 mm and the wall thickness is 1 mm. This ensures uniformity and ease of comparison across various tube configurations.

#### Numerical simulation

The energy absorption properties of circular tubes subjected to axial crush were meticulously investigated through the utilization of ABAQUS/Explicit (Abaqus 6.14, https://www.3ds.com/products-services/simulia/products/abaqus/) finite element method (FEM) software. In this analysis, the material properties were defined as those of steel in the previous experimental study as dipected in Appendix^8^. The Young's modulus of the steel is determined to be 205 GPa, with a Poisson's ratio of 0.28 and a yield stress of 233 MPa.

Two rigid plates were tied at the top and bottom edge of the tube and a friction coefficient of 0.2 applied as the general contact^11^. The top and bottom rigid plates have been completely constrained, except for the top plate, which is capable of downward movement to crush the tubes. A thorough mesh convergence analysis was conducted, leading to the selection of a mesh size of 5mm for the 1 mm thickness of S4R shell elements (see Appendix). It is noteworthy that the results are not mesh sensitive due to the folding-governed pattern of the helices.

As illustrated in Fig. 2b, the top rigid plate, meshed with R3D4 elements, was subjected to axial crushing against the tube at a controlled velocity of 2 m/s, following a smooth step amplitude curve. To ensure the validity of the quasi-static analysis, the focus is on the gradual deformation of materials, where the kinetic energy associated with the material's motion is considered small compared to its internal energy (typically 5–10%)^8^ (see Appendix).

The material parameters for the following two different cross-sections of the all BIDNATs and the conventional ones are set in the same way as for the circular cross-section.

#### Validation

The validation of the crush process for metallic tubes has been extensively studied in the literature, and this issue is no longer considered challenging in the present day. However, manufacturing BIDNAT, except by metal forming and welding process, poses a difficulty. In this regard, the authors of this study refer to a previously published paper that shares the same material properties but with different dimensions and boundary conditions for the purpose of validation. The experimental tests conducted in this paper validates the square tube cross-sections which has been demonstrated in the Appendix^8^.

### Elliptical and rectangular cross-section of the BIDNATs

**Figure 3:**
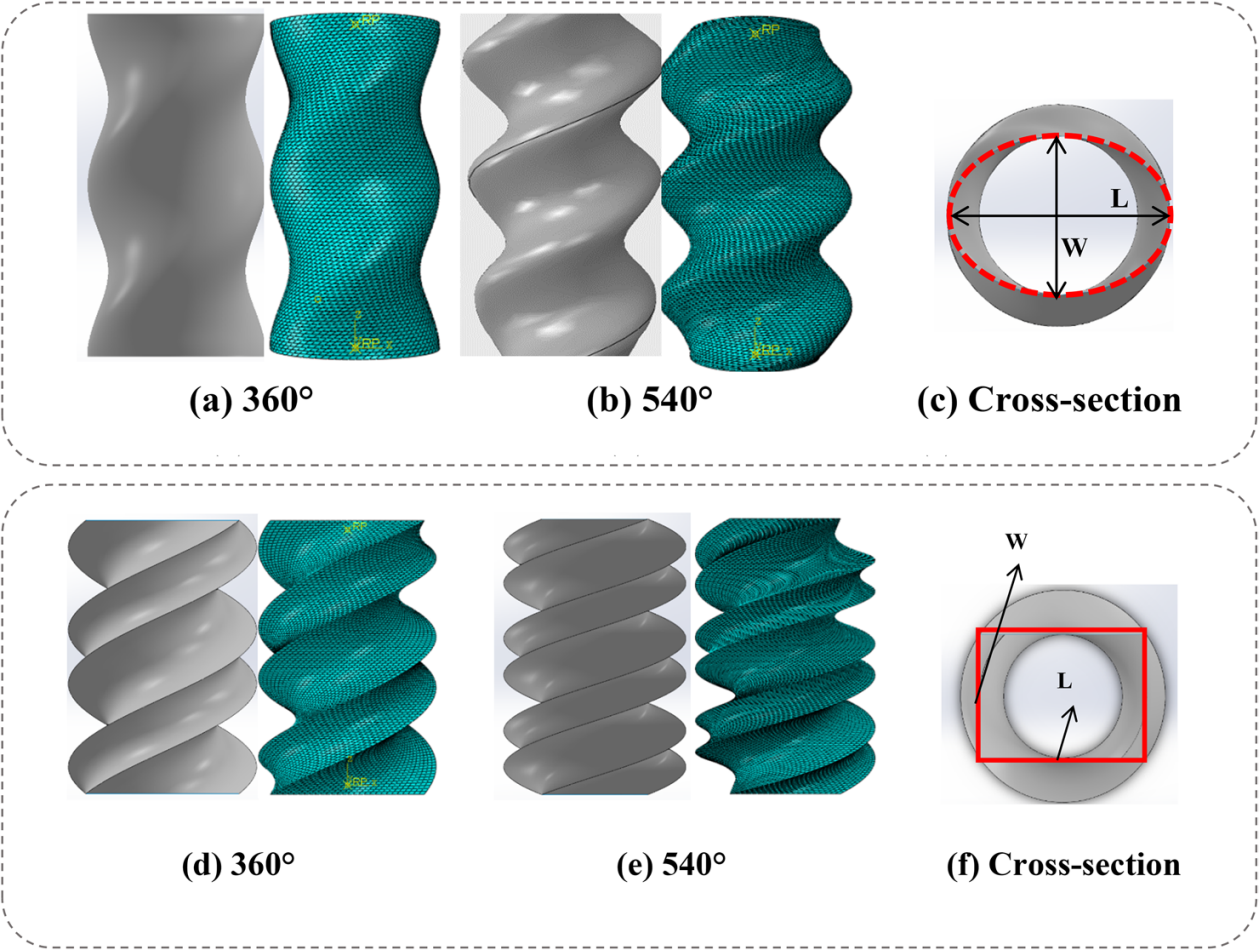
Dimensions and rotation angles of the BIDNAT, (**a**) the elliptical cross-section and (**b**) rectangular cross-section [created by Abaqus 6.14, (https://www.3ds.com/products-services/simulia/products/abaqus/) and SOLIDWORKS 2016 (www.solidworks.com)].

In Fig. 3, the graphical representation displays the BIDNAT characterized by the cross-sections of both elliptical and rectangular shapes. To examine the behavior and properties of these DNA structures, 18 distinct models were generated for elliptical and 18 for rectangular cross-sections with each model corresponding to a specific rotation angle of 360° or 540°. In addition 18 conventional elliptical and rectangular tubes have been simulated for comparison sake.

Those BIDNATs, featuring elliptical cross-sections, exhibit a helical shell configuration. Conversely, the BIDNAT with a rectangular cross-section displays a sharper outer edge on its shell, and the chiral shape of its shell is more prominently observed. This comprehensive analysis, involving the utilization of SolidWorks (SOLIDWORKS 2016, www.solidworks.com) software and incorporating the dimensional parameters outlined in Fig. 3, provides insight into the structural variations and features of BIDNATs with different cross-sections.

The nomenclature used to designate various BIDNATs follows a specific pattern. As an example, let's consider the name "E150/60." In this case, the prefix "E" indicates that the tube possesses an elliptical cross-sectional shape. The subsequent numbers, "150" and "60," represent the length and width, respectively, of the major and minor diameters of the elliptical cross-section.

To further elaborate on the nomenclature, when an elliptical cross-section is accompanied by a rotation angle of 540°, it is denoted by the prefix "AE," with the letter "A" signifying a rotation angle of 540° and when an elliptical cross-section is accompanied by non-rotational angle, conventional elliptical tube, it is denoted by the prefix "NE".

The identical nomenclature system is applied to the BIDNATs featuring rectangular cross-sections. In this case, the designation "R" signifies the presence of a rectangular shape, with the first number representing the length and the second number representing the width of the cross-section.

Furthermore, when a rectangular cross-section is subjected to a rotation angle of 540°, it is denoted by the prefix "AR," with the letter "A" indicating the 540° rotation angle and when an rectangular cross-section is accompanied by non-rotational angle, conventional rectangular tube, it is denoted by the prefix "NR".

Moreover, the energy absorption characteristics of all the specimens have been simulated using the same procedure by ABAQUS/Explicit (Abaqus 6.14, https://www.3ds.com/products-services/simulia/products/abaqus/) FEA. This model, originally developed for studying the energy absorption properties of circular cross-section tubes during axial crush, has been successfully adapted for investigating the behavior of BIDNATs with both elliptical and rectangular cross-sections.

## Results and discussions

In this section, we conduct a comprehensive analysis of the quasi-static crashworthiness of circular, elliptical, and rectangular cross-sections, along with DNA-inspired crush-tubes featuring different cross-sections. Our investigation centers on analyzing force and energy absorption displacement diagrams, while thoroughly examining the various modes of deformation. Through this systematic parameter study, our objective is to achieve a thorough and comprehensive understanding of the crashworthiness characteristics displayed by these tubes.

Equation 1 illustrates several key crashworthiness indicators, including energy absorption (EA), specific energy absorption (SEA), and mean crush force (MCF), respectively^48^.

1 $$EA = \int\limits_{l_1}^{l_2} {F(x).dx} ;\quad SEA = \frac{EA}{m};\quad MCF = \frac{{\int_{l_1}^{l_2} {F(x).dx} }}{{{l_2} - {l_1}}}$$

where $F(x)$ is reaction force, $m$ is the tube mass and ${l_1}$, ${l_2}$ are crushed displacements calculated from zero to the assigned points.

### Circular cross-sections

**Figure 4:**
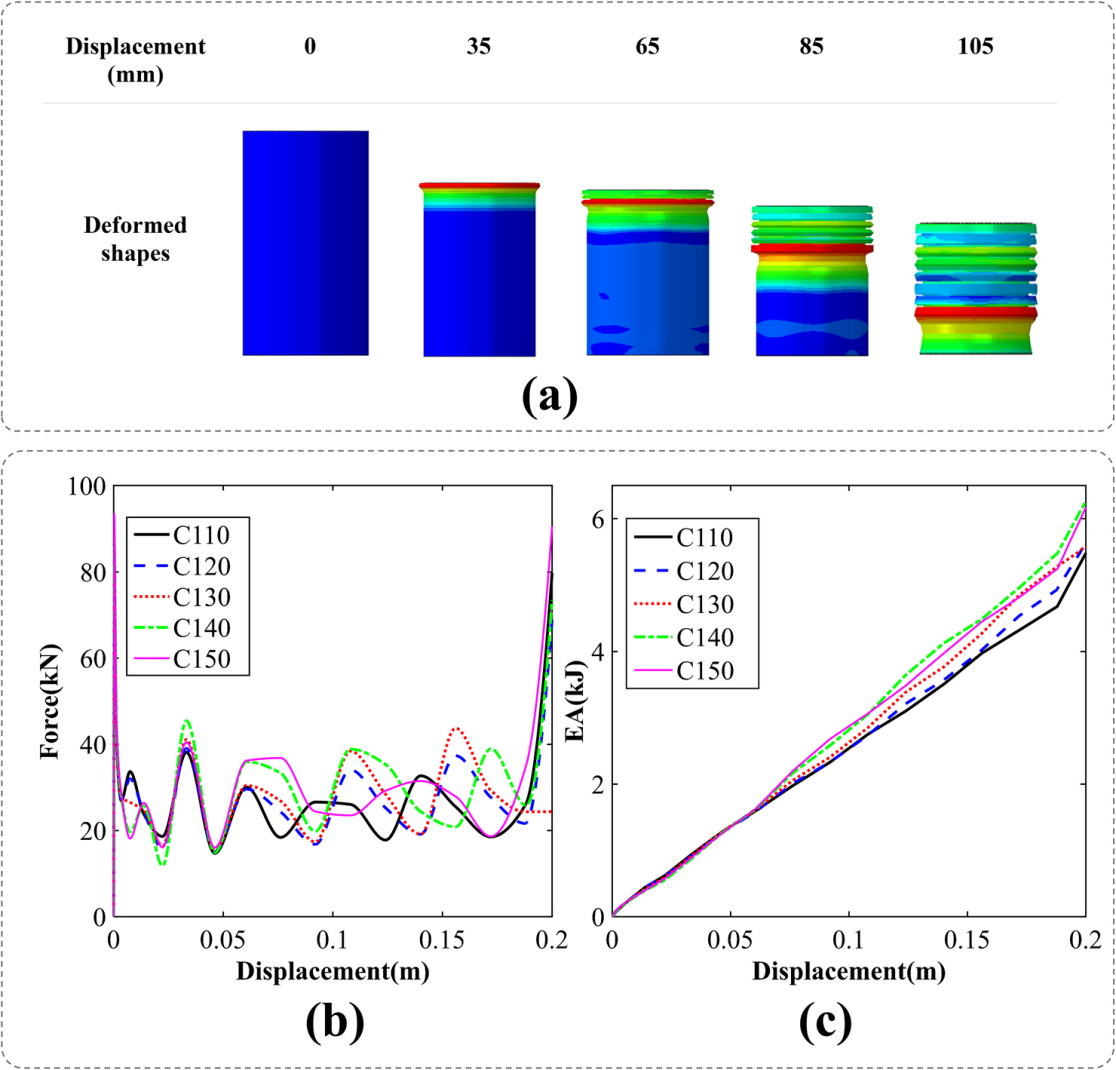
The axial crushing process of a cylindrical tubes, (**a**) deformation pattern [created by Abaqus 6.14, (https://www.3ds.com/products-services/simulia/products/abaqus/)], (**b**) force–displacement, (**c**) energy absorption-displacement curves.

Figure 4a depicts the axial crushing process of a cylindrical tube. The analysis focuses on the specific case of C110, revealing a gradual collapse of the cylinder wall, leading to the formation of a symmetrical and uniform shape. This symmetrical folding pattern demonstrates superior energy absorption efficiency compared to the formation of irregular folds during the crushing process. The findings highlight the advantageous nature of the observed symmetrical folding pattern in terms of energy absorption capabilities.

Figure 4b,c illustrate the force–displacement (F–D) and energy absorption-displacement (EA-D) curves derived from the axial crush simulation of five distinct circular cross-section tubes. Investigation of F–D curves reveals that C150 exhibits the highest peak force, reaching approximately 93 kN. The F–D curves exhibit fluctuations attributed to symmetrical folding, while the final stage of crushing remains almost identical for all cross-sections, except for C130.

At the immersion of each fold, the force undergoes an initial increase followed by a subsequent decrease until the fold is fully completed and the next fold is immersed. This pattern is observable in Fig. 4b, where each fold is depicted by of those fluctuations.

In Fig. 4c, the energy absorption-displacement values are presented for five distinct cross-circular sectional sizes. Notably, the final energy absorption value for C140 surpasses that of the other cross-sections. This observation suggests that the energy absorption capacity of circular tubes generally exhibits a positive correlation with the circular radius except C140 tube.

### Elliptical cross-sections

In this section the F–D and EA-D of the conventional and bio-inspired DNA tubes with elliptical cross-sections will be investigated.

#### Conventional elliptical cross-sections (NE)

**Figure 5:**
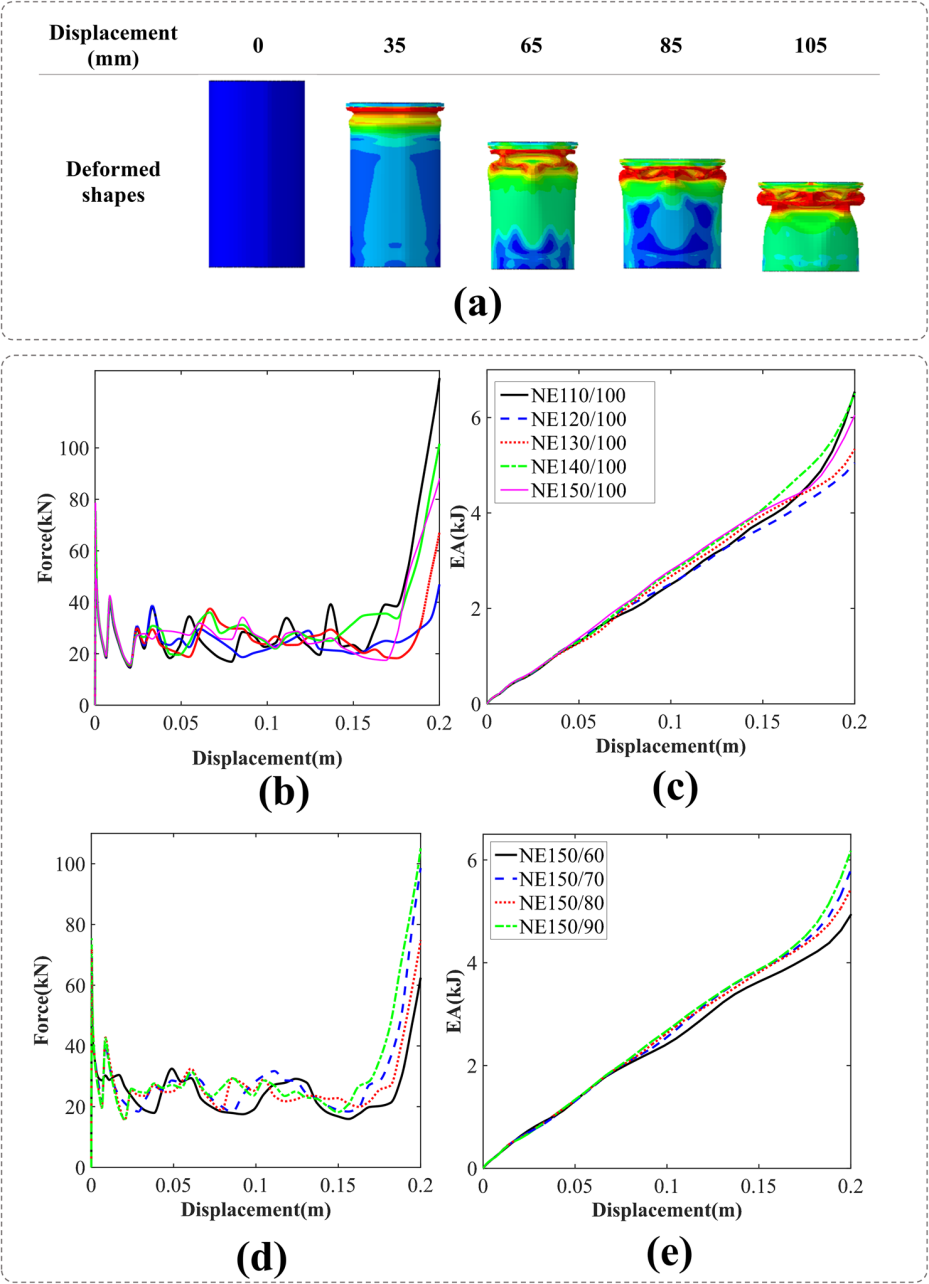
(**a**) Deformation process of a conventional elliptical cross-section tube with an aspect ratio of 110/100 [created by Abaqus 6.14, (https://www.3ds.com/products-services/simulia/products/abaqus/)], (**b**) force–displacement, (**c**) energy absorption-displacement of the conventional elliptical cross-section tubes with W = 100 mm, (**d**) force–displacement and (**e**) energy absorption-displacement of the conventional elliptical cross-section tubes with L = 150 mm.

In Fig. 5a, we examine the crushing process of the NE110/100 specimen. During the initial stage, the folding pattern exhibits symmetry. However, subsequent observations reveal a shift towards a dominant folding pattern characterized by diamond formations.

The F–D and EA-D of the conventional elliptical cross-section tubes are depicted in Fig. 5. In Fig. 5b, it is observed that the variation in the longest diameter of the ellipse does not have a discernible impact on the force–displacement curves during the initial stage of deformations.

Furthermore, it is noteworthy that the NE150/100 specimen exhibits the highest initial peak force, while the NE110/100 specimen demonstrates the highest final energy absorption. These results provide valuable insights into the distinctive characteristics and performance of these particular specimens. Similar to Fig. 4, each fluctuation corresponds to the formation of a fold. In the case of NE110/100, the first and second folds exhibit symmetry, while the subsequent folds display a diamond shape.

#### BIDNAT with the elliptical cross-sections (E)

**Figure 6:**
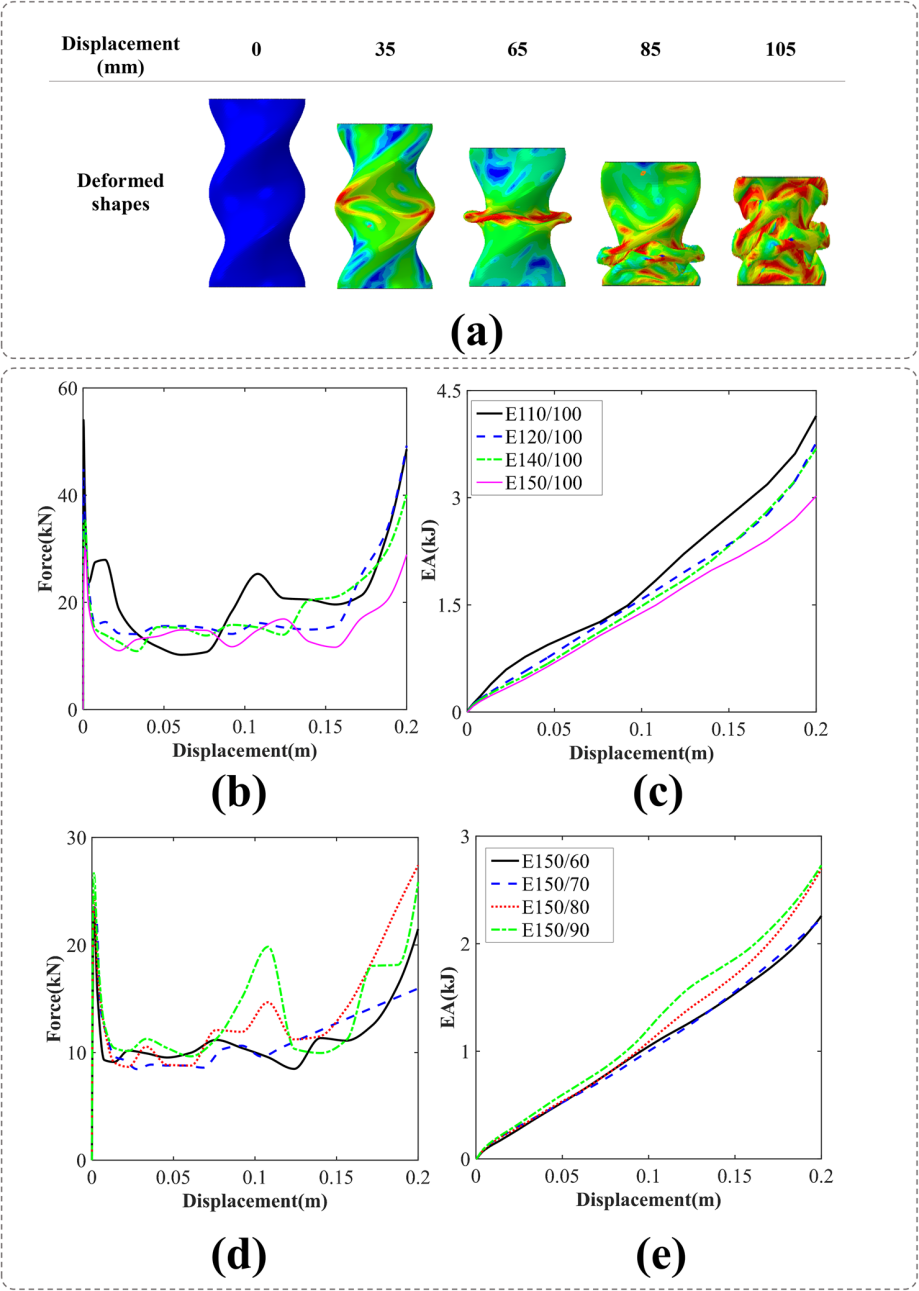
(**a**) the axial crushing process of the E150/100 BIDNAT tube [created by Abaqus 6.14, (https://www.3ds.com/products-services/simulia/products/abaqus/)], (**b**) force–displacement, (**c**) energy absorption-displacement of the elliptical cross-section BIDNATs, while the rotation angle of 360° and W = 100 mm, (**d**) force–displacement and e) energy absorption-displacement of the elliptical cross-section BIDNATs, while the rotation angle of 360° and L = 150 mm.

The technical analysis of the crushing simulation of the BIDNAT with an elliptical cross-section (E150/100) is depicted in Fig. 6a. The axial collision process reveals that the collapse pattern is influenced by the rotation angle of the cross-section. Under the downward force exerted by the rigid plate, the elliptical cross-section DNA shell undergoes significant deformation. As the crushing process advances, the shell accumulates in disordered layers. Notably, the number of shell layers increases in the case of the BIDNA elliptical section tube with the rotation angle of 540°.

The F–D and EA-D curves for various DNA-inspired elliptical cross-section aspect ratios with a rotation angle of 360° are illustrated in Fig. 6. It can be observed that increasing the larger diameter (L) of the ellipse leads to a decrease in energy absorption, while increasing the smaller diameter (W) results in an increase in energy absorption. Notably, the E110/100 configuration exhibits the highest IPF and EA due to its symmetrical cross-section and different folding patterns in comparison with other tubes.

#### BIDNAT with the elliptical cross-sections (AE)

**Figure 7:**
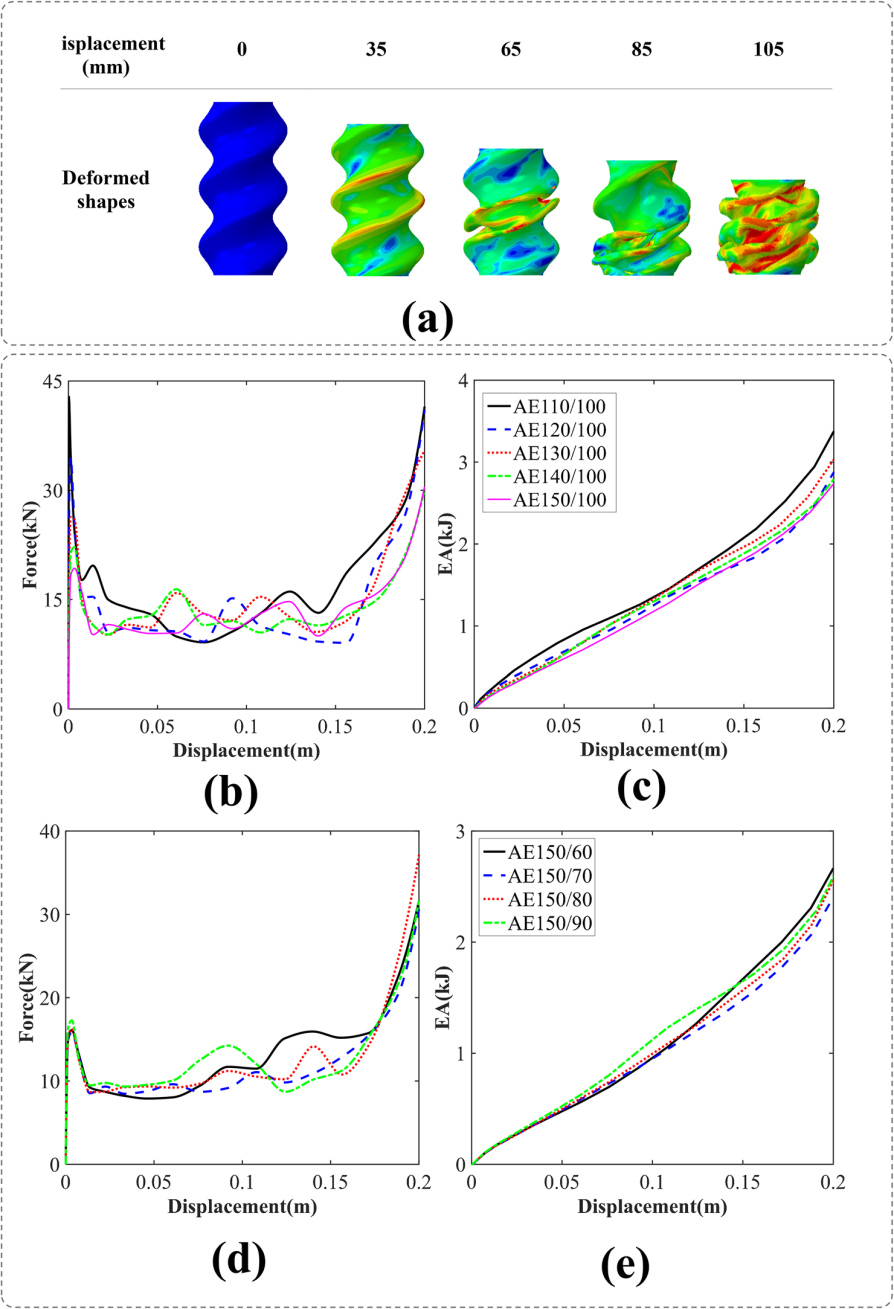
(**a**) The axial crushing process of the AE150/100 BIDNAT tube [created by Abaqus 6.14, (https://www.3ds.com/products-services/simulia/products/abaqus/)], (**b**) force–displacement, (**c**) energy absorption-displacement of the elliptical cross-section BIDNATs ( the rotation angle of 540° and W = 100 mm), (**d**) force–displacement and (**e**) energy absorption-displacement of the elliptical cross-section BIDNATs (the rotation angle of 540° and L = 150 mm).

The axial compression process of the AE150/100 BIDNAT, with the rotation angle of 540°, is depicted in Fig. 7a. The increased number of helices in the tube facilitates its collapse, resulting in lower IPF and smoother mean crushing forces (MCF) in comparison with the angle of 360°.

Among the specimens studied, the AE150/100 exhibits the highest IPF and final EA. Conversely, a significant reduction in IPF is observed when the larger diameter of the ellipse is increased, as depicted in Fig. 7. On the other hand, increasing the smaller diameter of the ellipse has a less pronounced effect on IPF, and the AE150/60 specimen achieves the highest final EA, as shown in Fig. 7d,e.

### Rectangular cross-sections

This section will examine the F–D and EA-D of both conventional and bio-inspired DNA tubes that have rectangular cross-sections.

#### Conventional rectangular cross-sections (NR)

**Figure 8:**
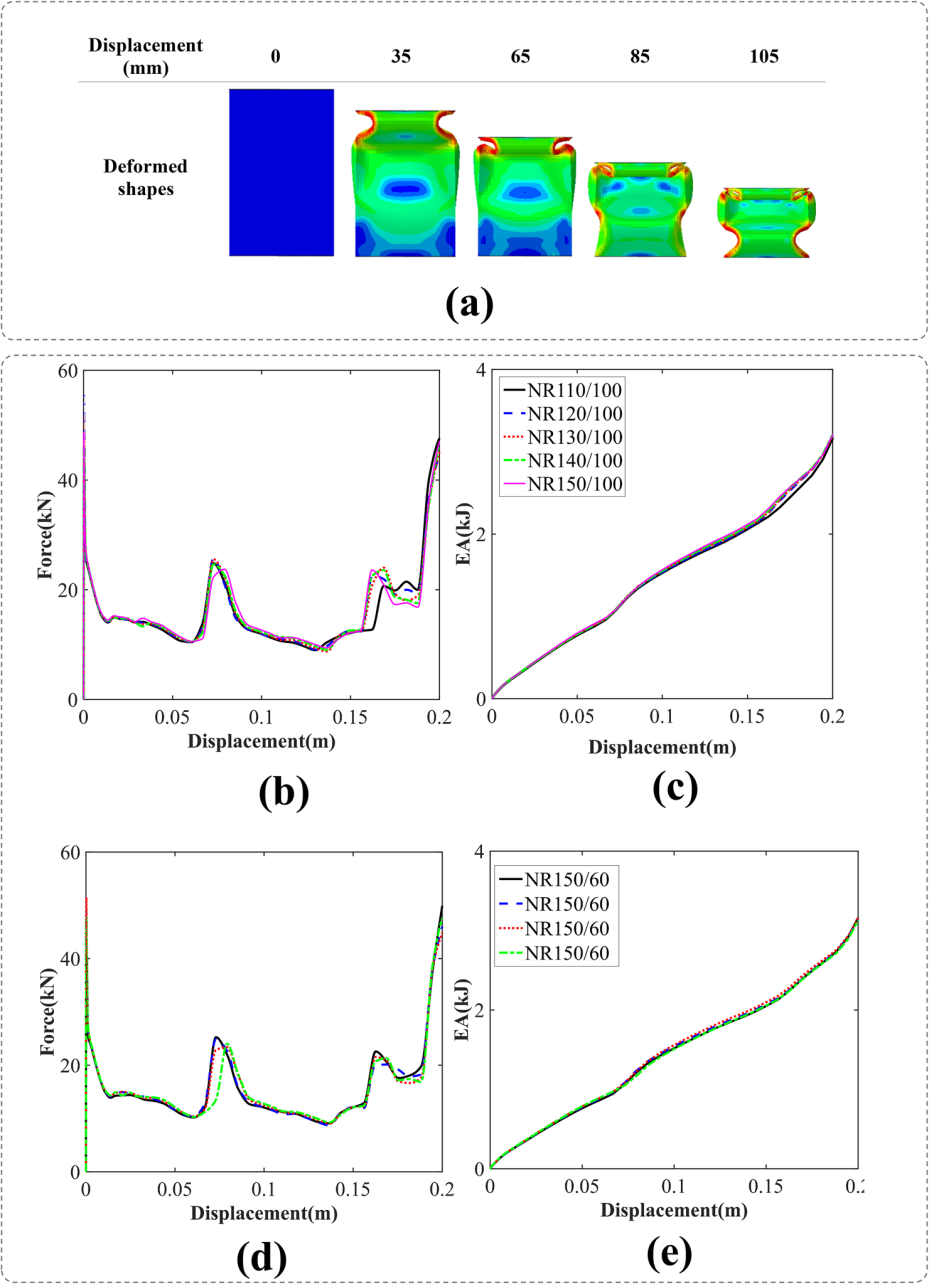
(**a**) The axial crushing process of the NR150/60 tube [created by Abaqus 6.14, (https://www.3ds.com/products-services/simulia/products/abaqus/)], (**b**) force–displacement, (**c**) energy absorption-displacement of the conventional rectangular cross-section tubes (W = 100 mm), (**d**) force–displacement, and e) energy absorption-displacement of the conventional rectangular cross-section tubes (L = 150 mm).

The folding process of the simple rectangular cross-section NR150/60 is illustrated in Fig. 8. The folding patterns exhibit regularity, with three distinct folds consistently observable across all the cross-sections.

It is evident that the EA of all rectangular specimens is nearly identical, as depicted in Fig. 8c,e; additionally, the highest IPF corresponds to the NR120/100 cross-section, as portrayed in Fig. 8b.

#### BIDNAT with the rectangular cross-sections (R)

**Figure 9:**
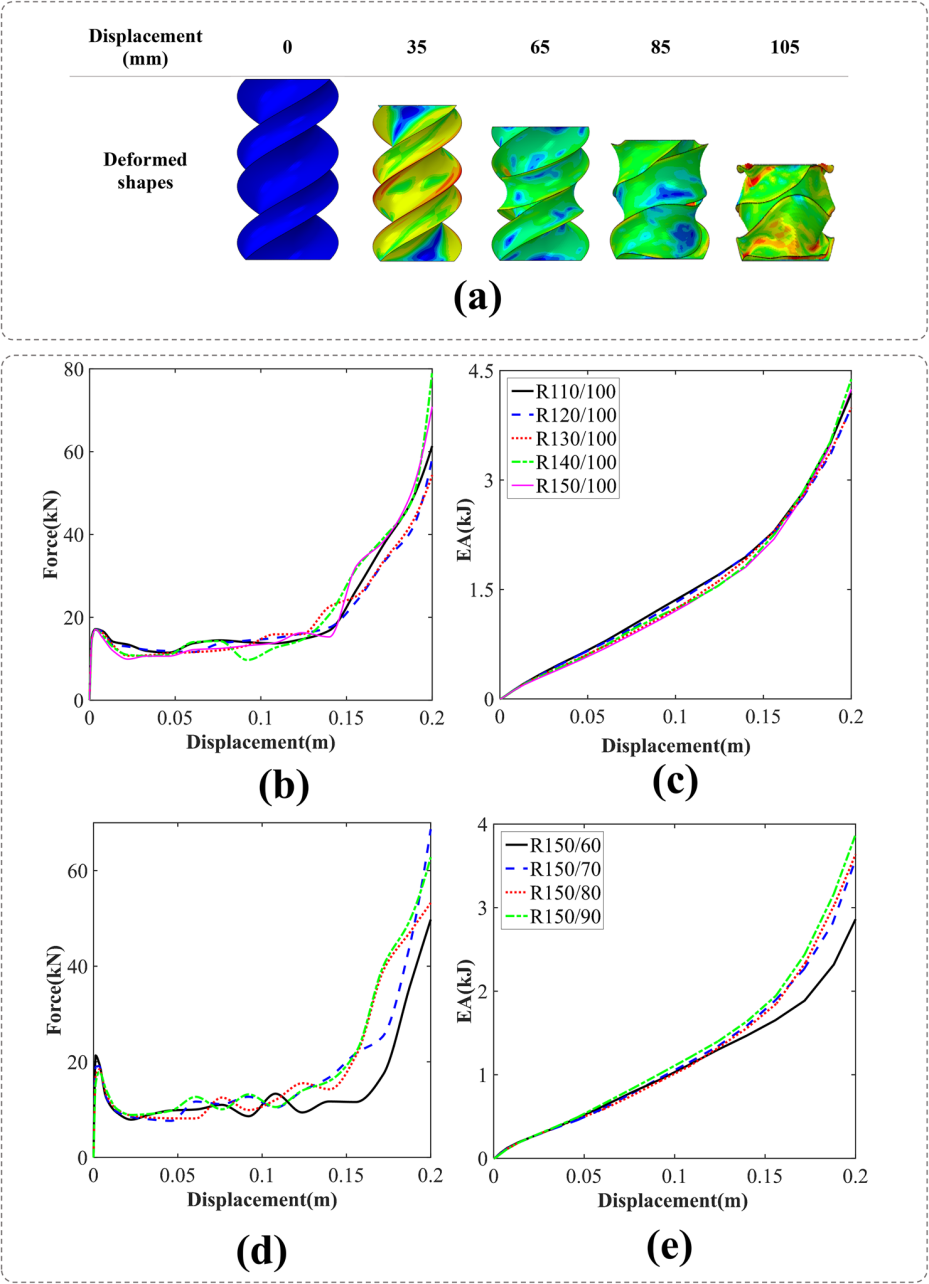
(**a**) The axial crushing process of the R150/100 BIDNAT tube [created by Abaqus 6.14, (https://www.3ds.com/products-services/simulia/products/abaqus/)], (**b**) force–displacement, (**c**) energy absorption-displacement of the rectangular cross-section BIDNATs (the rotation angle of 360° and L = 150 mm), (**d**) force–displacement and (**e**) energy absorption-displacement of the rectangular cross-section BIDNATs (the rotation angle of 360° and L = 150 mm).

The simulation process of DNA tube crushing with a rotation angle of 360° and the rectangular cross-section is shown in Fig. 9a. Taking R150/100, the deformation process as an example, its sharp edge helices have governed the crushing process and it will reduce the IPF for and smoother MCFs. The cross-sections with the width of 100 mm have shown lower IPF/MCF, which is desirable in crashworthiness characteristic of a structure (Fig. 9b). Moreover, the EA of them are the same through the crushing process in contrast to length 150 mm that have different EA at 0.2 mm crush length (Fig. 9c,e).

Based on the observed F–D curves presented in Figs. 9b,d, it is evident that the rectangular section of the bio-inspired DNA tubes with rotation angle of 360° has entered the third stage of energy absorption, which initiates at a deformation level of 0.15 mm.

#### BIDNAT with the rectangular cross-sections (AR)

**Figure 10:**
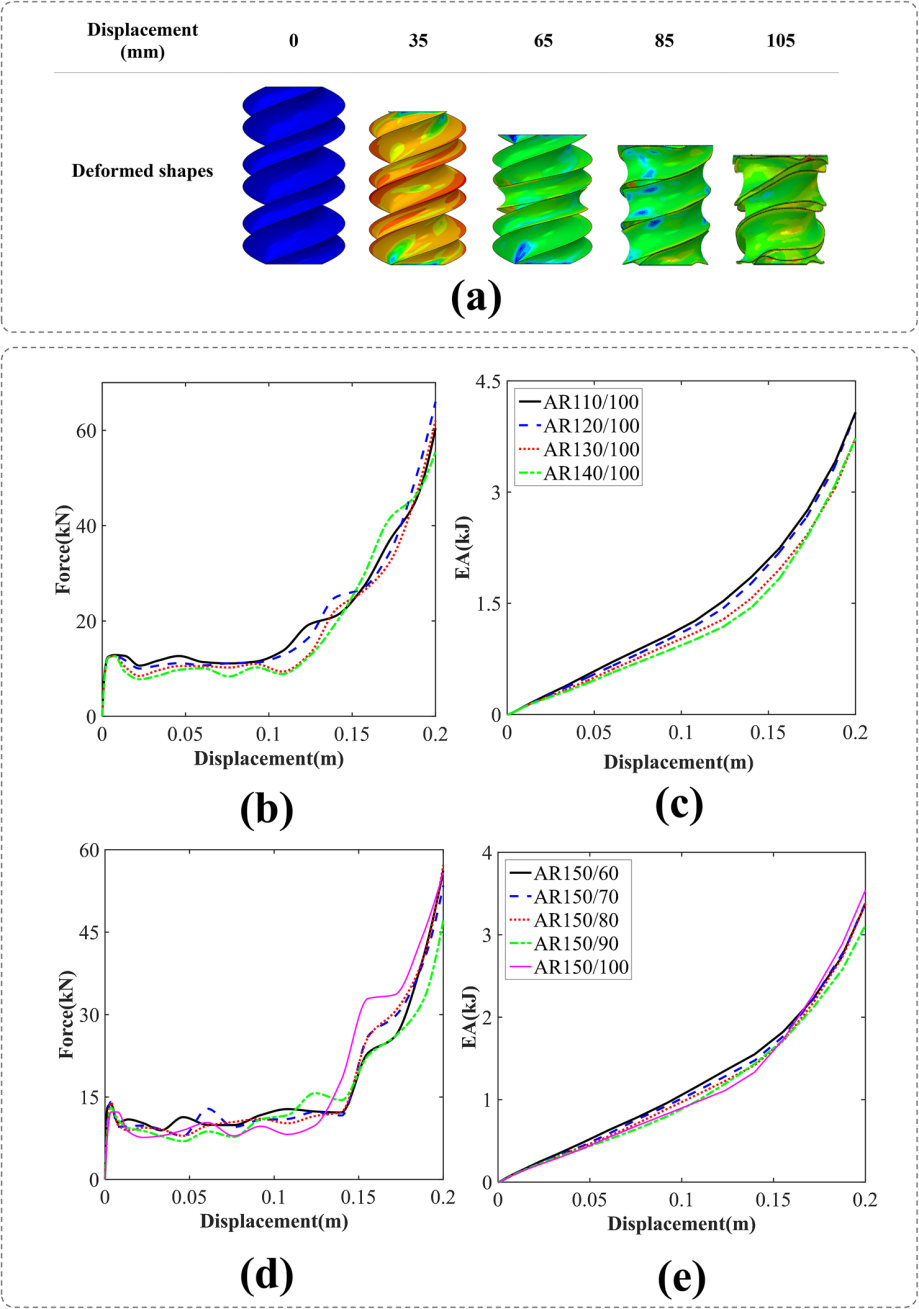
(**a**) The axial crushing process of the AR150/100 BIDNAT tube [created by Abaqus 6.14, (https://www.3ds.com/products-services/simulia/products/abaqus/)], (**b**) force–displacement, (**c**) energy absorption-displacement of the rectangular cross-section BIDNATs (the rotation angle of 540° and L = 150 mm), (**d**) force–displacement and (**e**) energy absorption-displacement of the rectangular cross-section BIDNATs (the rotation angle of 540° and L = 150 mm).

The simulation depicted in Fig. 10a shows the crushing of a DNA tube with the rotation angle of 540°, featuring a rectangular cross-section. Specifically, when considering the AR150/100 variant, the absence of distinct folds can be attributed to the local buckling of the shell. In this case, the crushing process is predominantly governed by the pre-fold pattern of the tubes. It is worth noting that this phenomenon is not limited to the AR150/100 cross-section but can also be observed in other cross-sectional configurations.

Figure 10b–e present the F–D and EA-D curves for the 540° rectangular cross-section BIDNATs. The tube exhibits constant width and varying length in one case (Fig. 10b,c), constant length and varying width in another (Fig. 10d,e).

The cross-sections with a width of 100 mm exhibit lower IPF/MCF in this bio-inspired structure, as seen in Fig. 10b. Furthermore, by analyzing the F–D curves in Fig. 10b,d, it becomes apparent that the rectangular cross-section of the BIDNATs, rotated at the angle of 540°, enters the third stage of energy absorption at lower crushing distance than the rotated angle of 360°.

### Comparison of crashworthiness of three cross-sections

The initial peak force and specific energy absorption are crucial parameters in evaluating the crashworthiness of crash tubes. The initial peak force represents the maximum force experienced by the tubes at the beginning of a crash event. It provides insight into the tubes' ability to withstand and distribute impact forces. Specific energy absorption, on the other hand, measures the amount of energy the tubes can absorb per unit of their mass (Eq. 1).

By considering these criteria, engineers and researchers can better understand the performance of crash tubes and design structures that can effectively absorb energy, protect occupants, and minimize structural damage during crash events.

**Figure 11:**
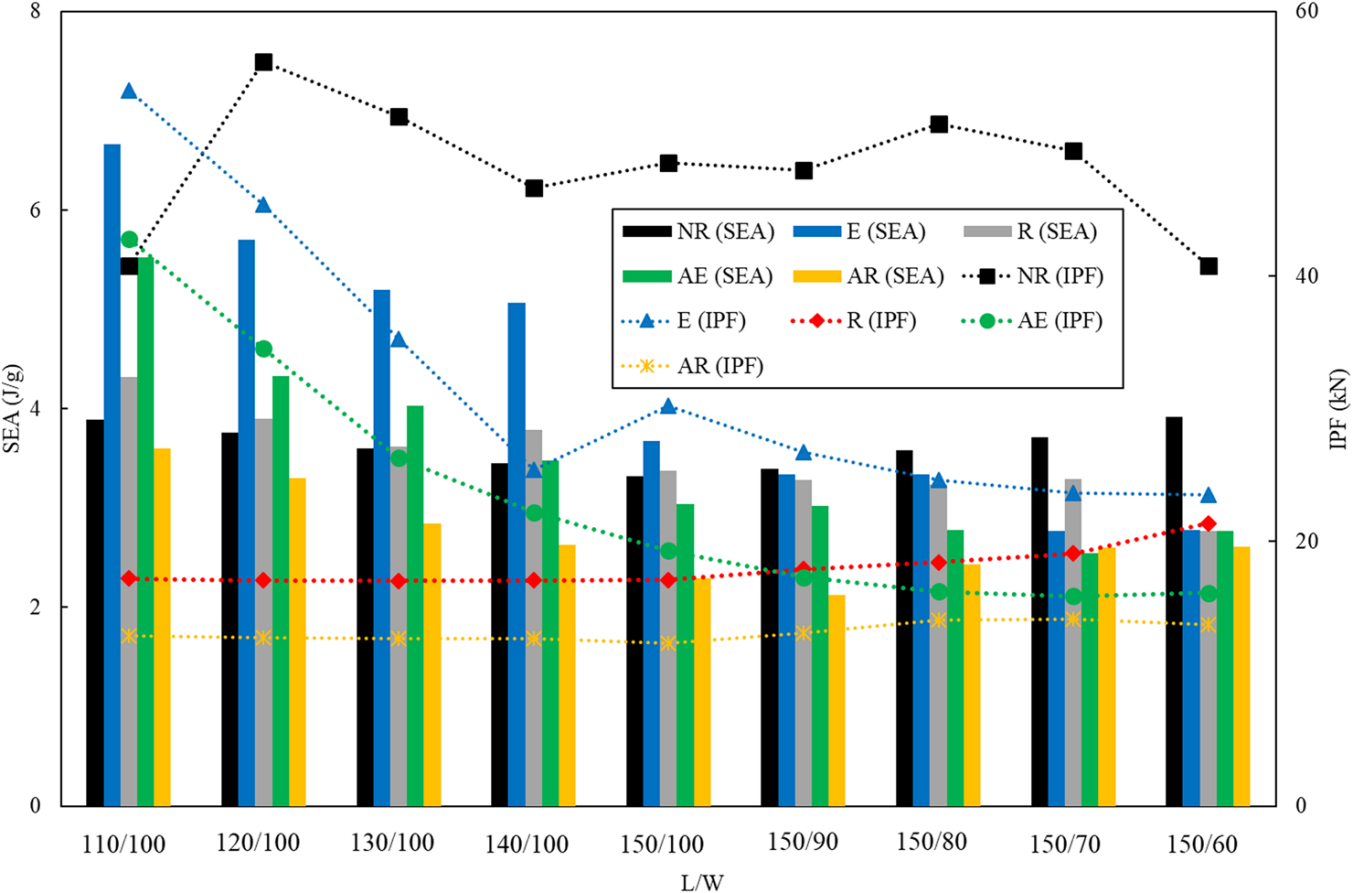
SEA and IPF comparison of the conventional rectangular cross-sections with BIDNATs.

Figure 11 presents a comparison between various BIDNATs and conventional rectangular cross-section tubes with identical length-to-width aspect ratios, focusing on the IPF and SEA. The increased rotation angle has led to a greater number of helices and a more pronounced helix angle relative to the tube's axis, resulting in a decrease in IPF. Apart from NR110/100, the lower cross-section aspect ratios (L/W) generally exhibit higher IPF values and higher SEA values.

As expected, the BIDNATs with rectangular cross-section and the rotation angle of 540° demonstrate the lowest SEA and IPF values across all aspect ratios. In certain aspect ratios like 150/90, 150/80, 150/70, and 150/60, the SEA value of the conventional rectangular cross-section tubes surpasses that of the BIDNATs, with an approximate 44% increase compared to the highest SEA in the 150/60 aspect ratio. Notably, for the 110/100 aspect ratio, the SEA of E110/100 is 71% higher than that of the conventional tube. Overall, the BIDNAT with elliptical cross-sections and the rotation angle of 360° exhibit higher SEA values and lower IPF values, particularly for W = 100 mm.

**Figure 12:**
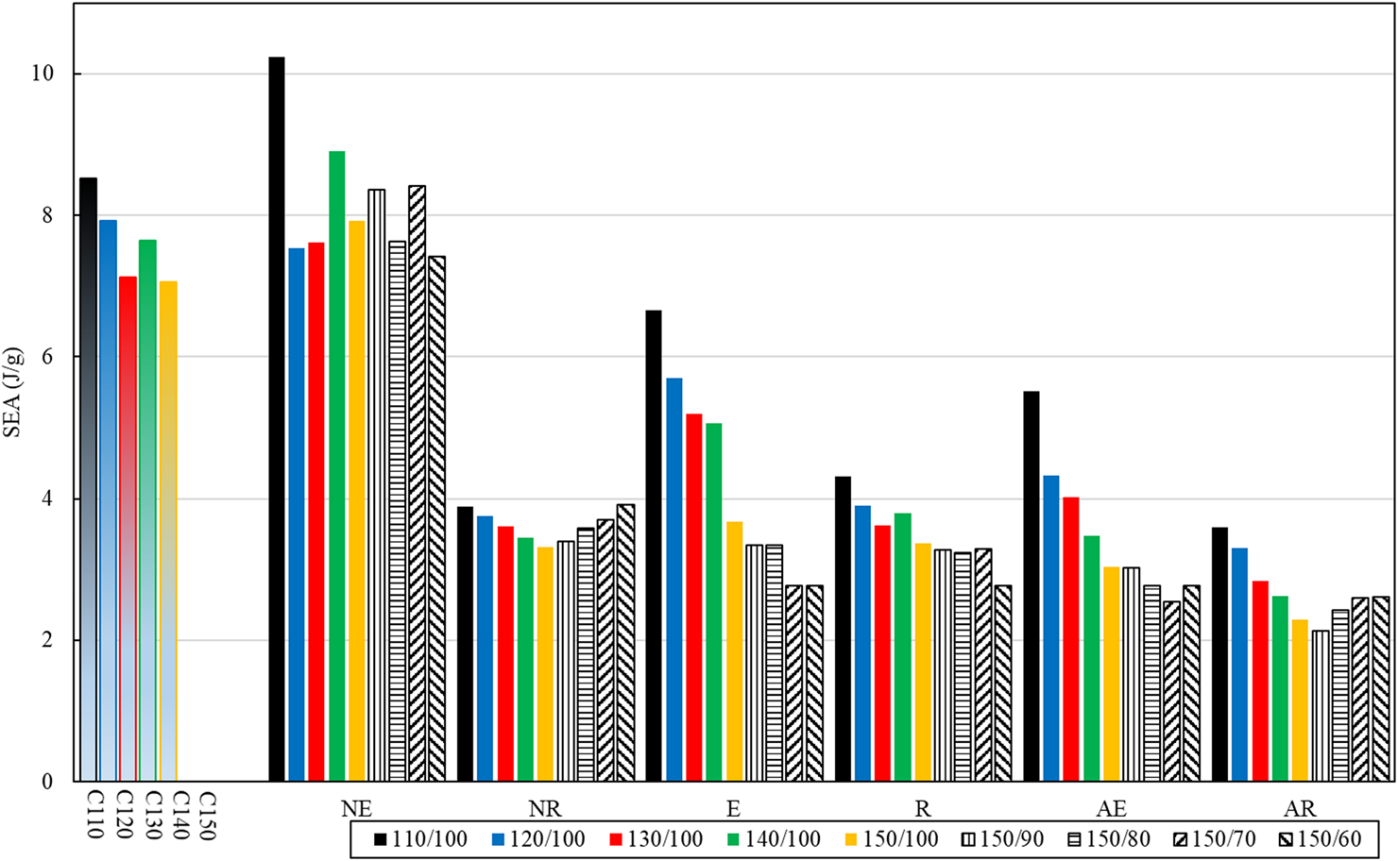
Comparison of the specific Energy Absorption (SEA) among all specimens.

**Figure 13:**
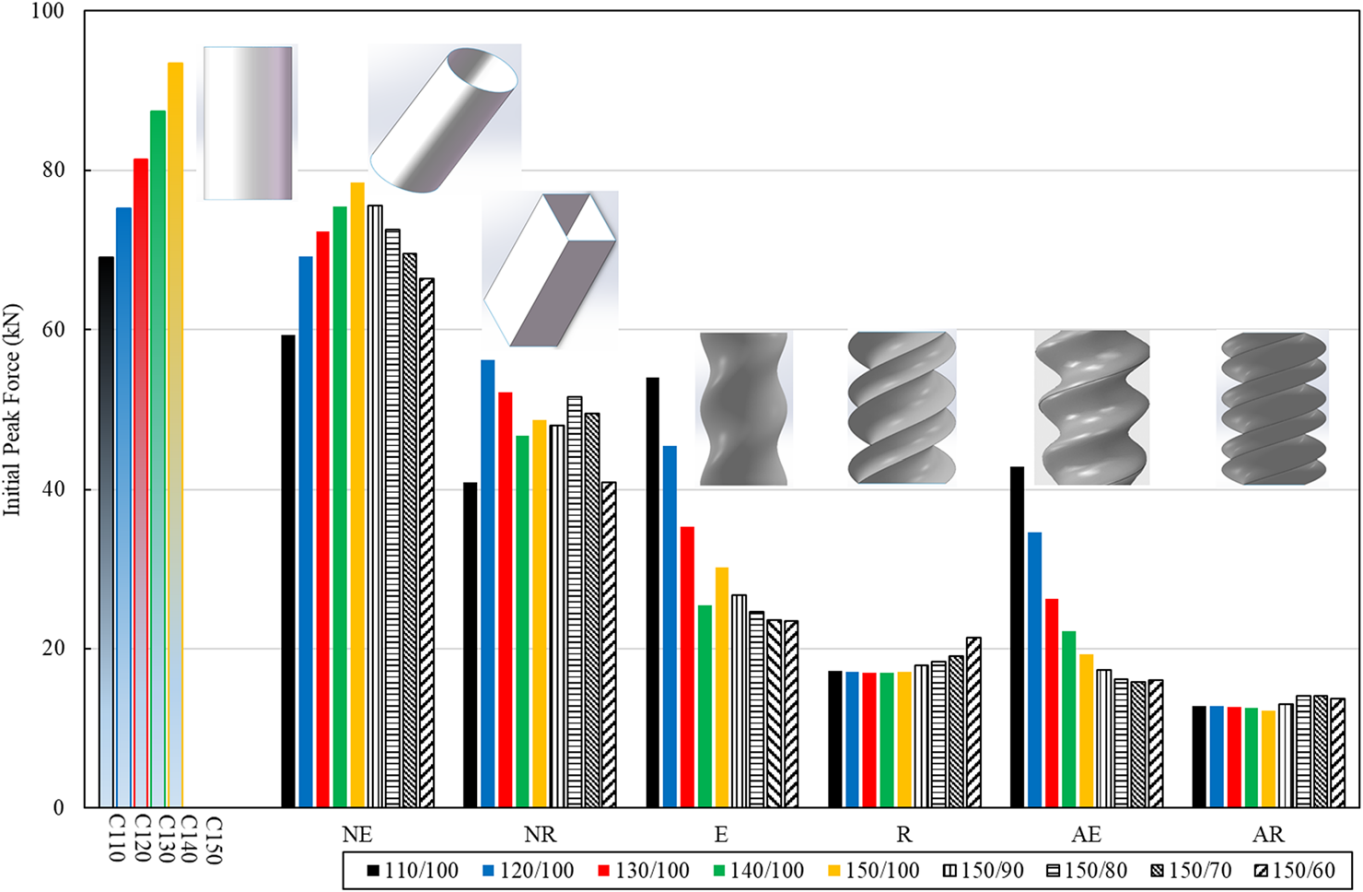
Comparison of the initial peak force (IPF) among all specimens.

The comparison between the SEA and IPF of various tubes is depicted in Figs. 12 and 13. These tubes consist of conventional circular, elliptical, and rectangular cross-sections, as well as BIDNA tubes. The conventional circular and elliptical tubes exhibit SEA values exceeding than 6 J/g, with only E110/100 surpassing this value among the DNA-inspired tubes. The NE110/100 tube boasts the highest SEA, surpassing E110/100 by 54%, while its IPF is 10% greater than the DNA-inspired E110/100. Notably, the conventional circular and elliptical cross-section tubes demonstrate higher IPF values when compared to the DNA-inspired counterparts.

### Discussion

**Figure 14:**
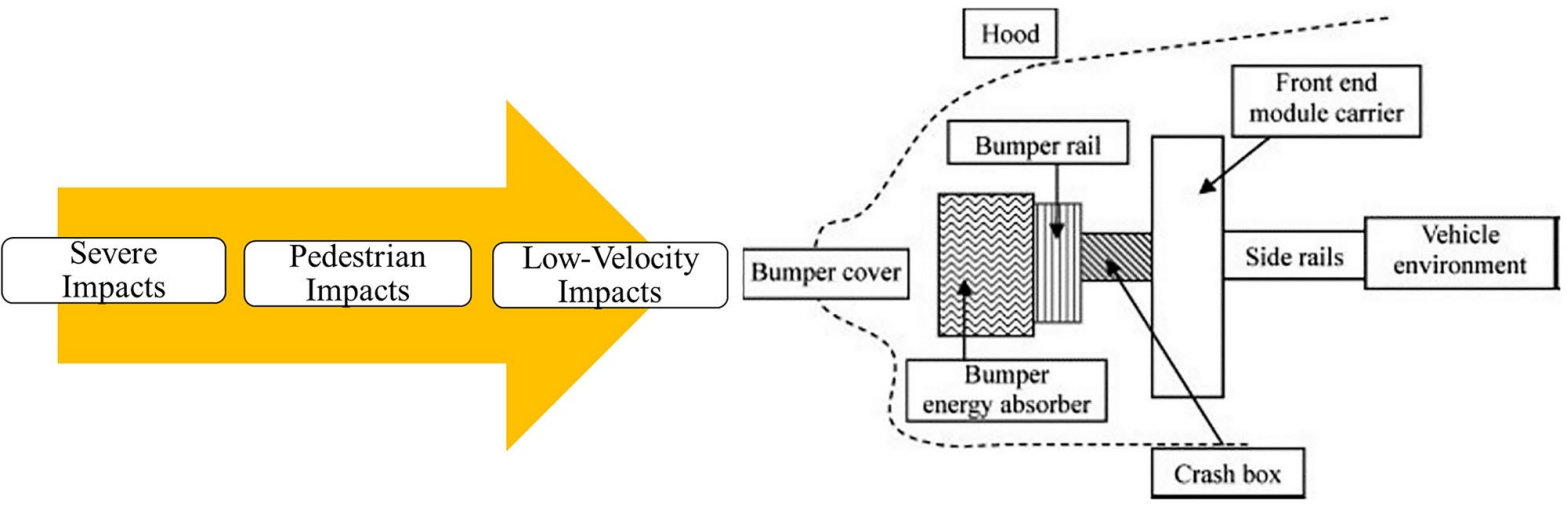
Various impact scenarios depicted with a schematic of the front-end of a passenger car.

Automotive crash boxes are vital components that enhance safety for vehicle occupants and pedestrians during collisions as demonstrated in Fig. 14. They absorb and dissipate impact energy, reducing forces transferred to occupants and minimizing injuries^60,61^.

#### Occupant safety

Crash boxes protect vehicle occupants during high-energy collisions by absorbing and distributing impact forces. They work alongside seat belts and airbags to mitigate the risk of head, chest, and lower extremity injuries^59^.

#### Pedestrian safety

Crash boxes are integrated into the front bumper area to reduce impact forces on pedestrians. They absorb energy and deform upon impact, minimizing leg injuries common in pedestrian accidents.

In recent times, the automotive industry has implemented more strict regulations regarding crash tests. These regulations have prompted automakers to enhance the structural integrity of the front end of vehicles, enabling them to effectively absorb and distribute impact forces. To meet these requirements, automakers have begun utilizing stiffer bumper beams and front cross members. However, it is equally crucial to consider the design of the front end with regards to pedestrian-leg-to-vehicle collisions, aiming to minimize the severity of injuries sustained by pedestrians. While the implementation of stiff bumper beams and crash boxes enhances the safety of vehicle occupants, they can exert significant forces on pedestrian legs. As a result, careful consideration must be given to the design of front crash boxes, highlighting the importance of the IPF.

This study introduces the concept of the BIDNAT as a potential solution for front crash boxes. Except for metal forming and welding procedures, one can consider hydroforming as one of the manufacturing methods for BIDNAT. The BIDNAT not only exhibits excellent energy absorption capabilities but also boasts a low IPF, making it a viable option. The SEA and IPF of BIDNATs can be controlled by adjusting parameters such as graded helix pitches, graded thicknesses and designate them in series^8^, offering flexibility in design and performance optimization.

## Conclusions

This paper provides a comprehensive FE-simulation of the energy absorption properties of bio-inspired DNA thin-walled tubes with circular, elliptical, and rectangular cross-sections. The analysis focuses on investigating deformation modes, force–displacement curves, and energy absorption capabilities through experiments and simulations. Key findings from the study include:

- Bio-inspired DNA tubes (BIDNATs) with elliptical and rectangular cross-sections showed lower initial peak forces and mean crushing forces compared to conventional tubes due to their helical geometry and sharp edges.
- BIDNATs with 360° rotation angles exhibited better energy absorption performance than those with 540° rotation angles.
- Among the specimens studied, the E110/100 BIDNAT demonstrated the highest initial peak force and final energy absorption.
- While conventional circular and elliptical tubes showed higher specific energy absorption compared to most BIDNATs, the E110/100 BIDNAT achieved 71% higher specific energy absorption than the conventional rectangular tube with the same aspect ratio.
- In summary, BIDNATs show potential as effective energy absorbers for applications like automotive crash boxes due to their tunable crashworthiness properties and relatively low initial peak forces, which can minimize pedestrian injuries. However, further optimizations are still needed to improve their energy absorption performance.

### Supplementary Information

Supplementary Information.

## Data Availability

The datasets used and/or analyzed during the current study available from the corresponding author on reasonable request.

## References

[1] Sun G (2017). Parameterization of criss-cross configurations for multiobjective crashworthiness optimization. Int. J. Mech. Sci..

[2] Baroutaji A, Sajjia M, Olabi A-G (2017). On the crashworthiness performance of thin-walled energy absorbers: Recent advances and future developments. Thin-Walled Struct..

[3] Sun G (2021). Parallelized optimization design of bumper systems under multiple low-speed impact loads. Thin-Walled Struct..

[4] Sun G (2022). Lightweight hybrid materials and structures for energy absorption: A state-of-the-art review and outlook. Thin-Walled Struct..

[5] Salehghaffari S (2010). Attempts to improve energy absorption characteristics of circular metal tubes subjected to axial loading. Thin-Walled Struct..

[6] Singace AA, El-Sobky H (1997). Behaviour of axially crushed corrugated tubes. Int. J. Mech. Sci..

[7] Eyvazian A (2014). Axial crushing behavior and energy absorption efficiency of corrugated tubes. Mater. Des..

[8] Shojaeefard MH (2014). Experimental and numerical crashworthiness investigation of combined circular and square sections. J. Mech. Sci. Technol..

[9] Najibi A, Shojaeefard MH, Yeganeh M (2016). Developing and multi-objective optimization of a combined energy absorber structure using polynomial neural networks and evolutionary algorithms. Latin Am. J. Solids Struct..

[10] Sun G (2017). An experimental and numerical study on quasi-static and dynamic crashing behaviors for tailor rolled blank (TRB) structures. Mater. Des..

[11] Aghamirzaie M, Najibi A, Ghasemi-Ghalebahman A (2023). Energy absorption investigation of octagonal multi-layered origami thin-walled tubes under quasi-static axial loading. Int. J. Crashworthiness.

[12] Ma J, You Z (2014). Energy absorption of thin-walled square tubes with a prefolded origami pattern—Part I: Geometry and numerical simulation. J. Appl. Mech..

[13] Alomarah A, Masood SH, Ruan D (2022). Dynamic and quasistatic properties of an auxetic structure: A comparative study. Adv. Eng. Mater..

[14] Lee W (2019). Effect of auxetic structures on crash behavior of cylindrical tube. Compos. Struct..

[15] Alomarah A, Al-Ibraheemi ZA, Ruan D (2023). 3D printed auxetic stents with re-entrant and chiral topologies. Smart Mater. Struct..

[16] Alexander JM (1960). An approximate analysis of the collapse of thin cylindrical shells under axial loading. Q. J. Mech. Appl. Math..

[17] Pugsley A (1960). The large-scale crumpling of thin cylindrical columns. Q. J. Mech. Appl. Math..

[18] Grzebieta RH (1990). An alternative method for determining the behaviour of round stocky tubes subjected to an axial crush load. Thin-Walled Struct..

[19] Wierzbicki T (1992). Alexander revisited—A two folding elements model of progressive crushing of tubes. Int. J. Solids Struct..

[20] Singace AA, Elsobky H, Reddy TY (1995). On the eccentricity factor in the progressive crushing of tubes. Int. J. Solids Struct..

[21] Abramowicz W, Jones N (1986). Dynamic progressive buckling of circular and square tubes. Int. J. Impact Eng.

[22] Abramowicz W, Wierzbicki T (1989). Axial crushing of multicorner sheet metal columns. ASME. J. Appl. Mech..

[23] Tang Z, Liu S, Zhang Z (2012). Energy absorption properties of non-convex multi-corner thin-walled columns. Thin-Walled Struct..

[24] Li Y, You Z (2019). Origami concave tubes for energy absorption. Int. J. Solids Struct..

[25] Li Q (2023). Axial crashworthiness design of double-hat beams with various cross-sections. Eng. Struct..

[26] Jones N (2010). Energy-absorbing effectiveness factor. Int. J. Impact Eng..

[27] Wu S (2016). On design of multi-cell thin-wall structures for crashworthiness. Int. J. Impact Eng.

[28] Qiu N (2016). Theoretical prediction and optimization of multi-cell hexagonal tubes under axial crashing. Thin-Walled Struct..

[29] Xiang Y, Yu T, Yang L (2016). Comparative analysis of energy absorption capacity of polygonal tubes, multi-cell tubes and honeycombs by utilizing key performance indicators. Mater. Des..

[30] Zhang X, Zhang H (2015). Some problems on the axial crushing of multi-cells. Int. J. Mech. Sci..

[31] Zhang X, Zhang H (2015). The crush resistance of four-panel angle elements. Int. J. Impact Eng.

[32] Sun G (2014). Crashing analysis and multiobjective optimization for thin-walled structures with functionally graded thickness. Int. J. Impact Eng.

[33] Zhang X, Wen Z, Zhang H (2014). Axial crushing and optimal design of square tubes with graded thickness. Thin-Walled Struct..

[34] Fang J (2015). Dynamic crashing behavior of new extrudable multi-cell tubes with a functionally graded thickness. Int. J. Mech. Sci..

[35] Zheng G (2016). Theoretical, numerical, and experimental study on laterally variable thickness (LVT) multi-cell tubes for crashworthiness. Int. J. Mech. Sci..

[36] Pang T (2019). Energy absorption mechanism of axially-varying thickness (AVT) multicell thin-walled structures under out-of-plane loading. Eng. Struct..

[37] San Ha N (2021). Energy absorption characteristics of bio-inspired hierarchical multi-cell square tubes under axial crushing. Int. J. Mech. Sci..

[38] Najibi A, Ghazifard P, Alizadeh P (2021). Numerical crashworthiness analysis of a novel functionally graded foam-filled tube. J. Sandwich Struct. Mater..

[39] Wang E (2021). On multiaxial failure behavior of closed-cell aluminum foams under medium strain rates. Thin-Walled Struct..

[40] Yu X (2022). Low-velocity impact of density-graded foam-filled square columns. Int. J. Crashworthiness.

[41] Wang Y-J, Zhang Z-J, Feng R-X (2023). Effect of plate-lattice structural material filling on crashworthiness performance of square tube. Mater. Lett..

[42] Hao P, Du J (2018). Energy absorption characteristics of bio-inspired honeycomb column thin-walled structure under impact loading. J. Mech. Behav. Biomed. Mater..

[43] Lazarus BS (2020). A review of impact resistant biological and bioinspired materials and structures. J. Mater. Res. Technol..

[44] Alomarah A, Yuan Y, Ruan D (2023). A bio-inspired auxetic metamaterial with two plateau regimes: Compressive properties and energy absorption. Thin-Walled Struct..

[45] Liu Q (2017). Energy absorption of bio-inspired multi-cell CFRP and aluminum square tubes. Compos. Part B Eng..

[46] Huang F (2023). Crashworthiness analysis of bio-inspired hierarchical circular tube under axial crushing. J. Mater. Sci..

[47] Hu D (2019). Energy-absorption characteristics of a bionic honeycomb tubular nested structure inspired by bamboo under axial crushing. Compos. Part B Eng..

[48] Deng X, Qin S, Huang J (2022). Crashworthiness analysis of gradient hierarchical multicellular columns evolved from the spatial folding. Mater. Des..

[49] Gong C (2020). Crashworthiness analysis of bionic thin-walled tubes inspired by the evolution laws of plant stems. Thin-Walled Struct..

[50] Chen Y (2023). Crashworthiness of bionic tree-shaped hexagonal hierarchical gradient structures under oblique crushing conditions. Mech. Adv. Mater. Struct..

[51] Chen J (2015). Review of beetle forewing structures and their biomimetic applications in China:(I) On the structural colors and the vertical and horizontal cross-sectional structures. Mater. Sci. Eng. C.

[52] Jiang B (2019). Numerical, theoretical, and experimental studies on the energy absorption of the thin-walled structures with bio-inspired constituent element. Int. J. Mech. Sci..

[53] Xiang X (2020). Energy absorption of bio-inspired multi-layered graded foam-filled structures under axial crushing. Compos. Part B Eng..

[54] Nikkhah H (2020). Evaluation of crushing and energy absorption characteristics of bio-inspired nested structures. Thin-Walled Struct..

[55] Wang C (2018). Structure design and multi-objective optimization of a novel crash box based on biomimetic structure. Int. J. Mech. Sci..

[56] Yao R (2022). A bio-inspired foam-filled multi-cell structural configuration for energy absorption. Compos. Part B Eng..

[57] Xi C, Najibi A, Zheng D (2019). Lateral energy absorption analysis of a new bioinspired DNA lattice structure. Iran. J. Sci. Technol. Trans. Mech. Eng..

[58] Zheng B (2019). Novel mechanical behaviors of DNA-inspired helical structures with chirality. Int. J. Mech. Sci..

[59] Najibi A, Ghazifard P, Torkian J (2024). On the crashworthiness optimisation of a new multi-corner tube under axial loading. Ships Offshore Struct..

[60] Mortazavi Moghaddam A, Kheradpisheh A, Asgari M (2021). A basic design for automotive crash boxes using an efficient corrugated conical tube. Proc. Inst. Mech. Eng. Part D J. Automob. Eng..

[61] Yusof NSB (2020). Materials selection of “green” natural fibers in polymer composite automotive crash box using DMAIC approach in Six Sigma method. J. Eng. Fibers Fabrics.

