# Supplementary material for: On the crashworthiness analysis of bio-inspired DNA tubes
Source: Sci Rep. 2024 Apr 12;14:8531. doi: 10.1038/s41598-024-59258-2 (PMC11014913; doi:10.1038/s41598-024-59258-2)
Supplement: Supplementary file 1 — Supplementary Information. [file 41598_2024_59258_MOESM1_ESM.docx]

**Appendix**

Figure A1 demonstrates that the kinetic energy throughout the entire process is less than 1% of the internal energy, which ensures a quasi-static process.

The data presented in figure A2 illustrates the force and energy absorption displacement of a rectangular cross-section tube. The difference between the two plots is attributed to the boundary conditions, which were defined as "tie" in the simulation. In the experiment, the top and bottom edges of the tube were constrained by fixtures and bolts to mimic a tie constraint. However, there is approximately a 5% difference in total energy absorption between the simulation and the experimental results, and the IPF differences also stand at 5%. This discrepancy indicates the level of agreement between the results.


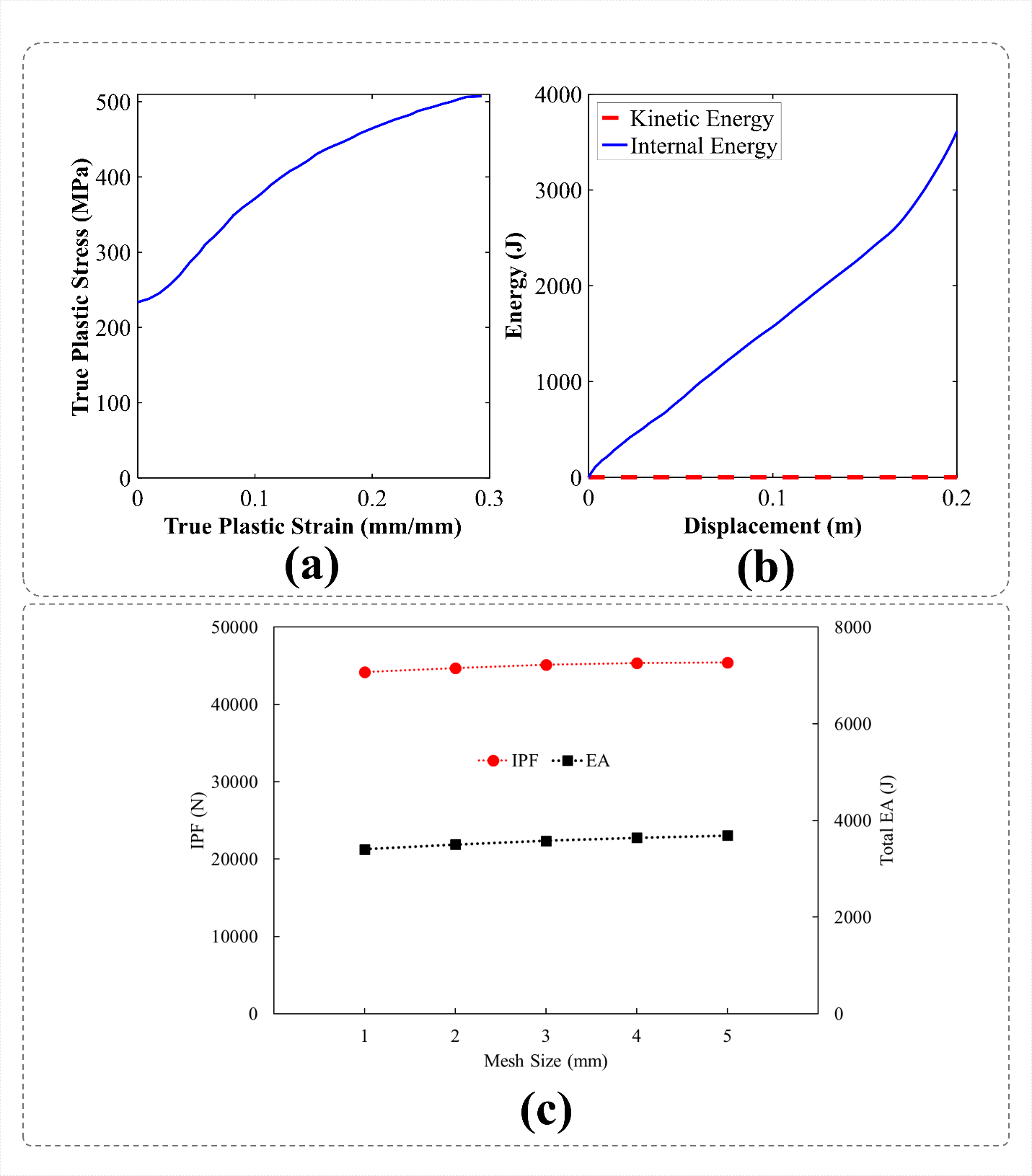
Figure A1: FEM simulation material properties, quasi-static assessment and mesh sensitivity analysis. a) True plastic stress and strain curve [8], b) internal and kinetic energy comparison and c) mesh sensitivity analysis for IPF and total EA of E120/110 specimen.


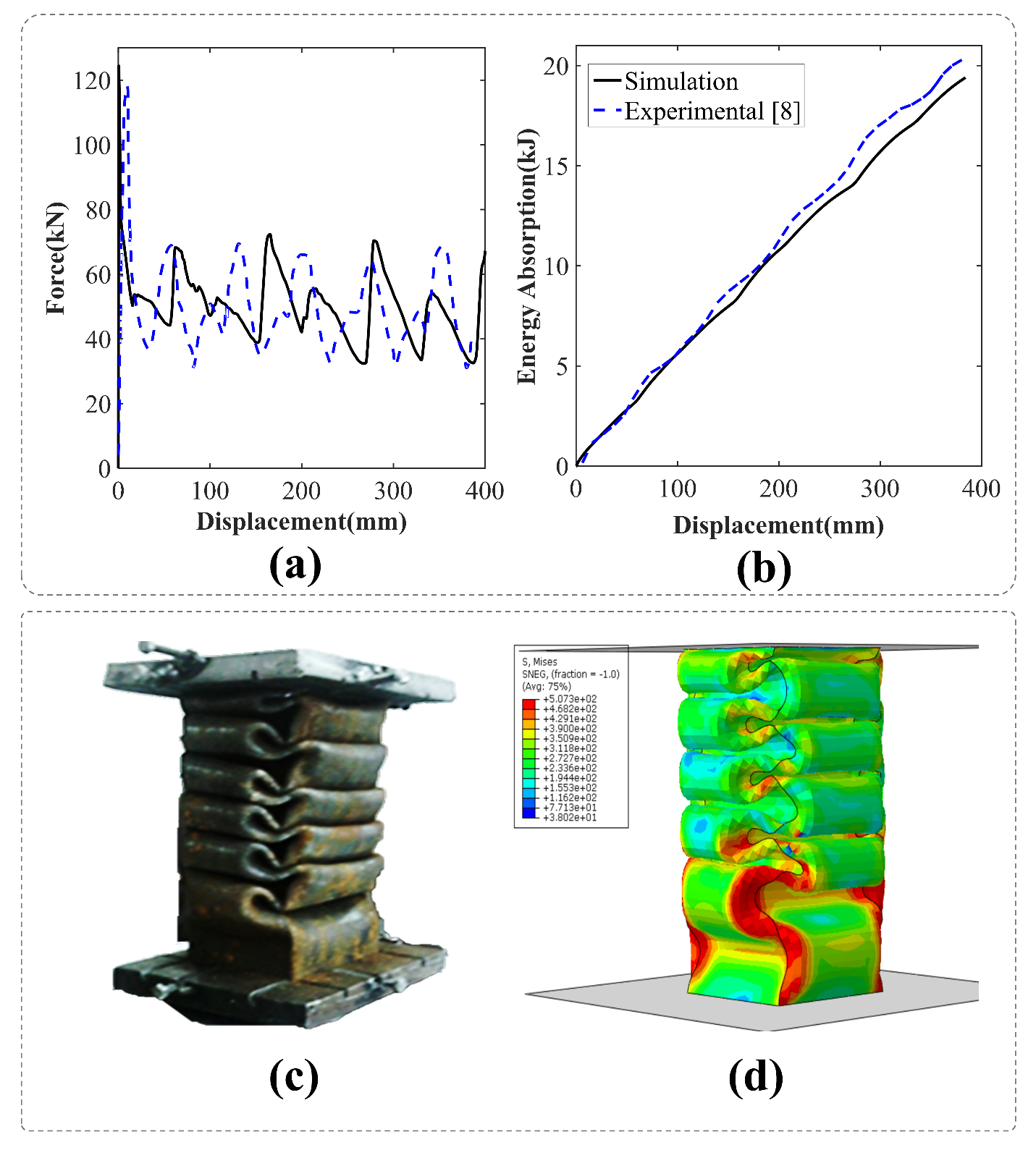


Figure A2. Experimental validation of the square cross-section tube with the dimensions of $90\times90\times588$ mm and the thickness of 2 mm. a) Force-displacement curves, b) energy absorption -displacement curves, c) experimental crushed tube and d) numerical crushed tube.
